# Prognostic effect of lncRNA BBOX1-AS1 in malignancies: a meta-analysis

**DOI:** 10.3389/fgene.2023.1234040

**Published:** 2023-08-11

**Authors:** Guangyao Lin, Yongzhou Wang, Li Deng, Tao Ye

**Affiliations:** ^1^ Department of Gynecology, Longhua Hospital, Shanghai University of Traditional Chinese Medicine, Shanghai, China; ^2^ Department of Gynecology, The Affiliated Traditional Chinese Medicine Hospital of Southwest Medical University, Luzhou, China

**Keywords:** lncRNA BBOX1-AS, human malignancies, meta-analysis, prognosis, review

## Abstract

**Background:** With the increasing number of new cancer cases and mortality rates, cancer has become a serious global health problem, but there are no ideal cancer biomarkers for effective diagnosis. Currently, mounting evidence demonstrates that lncRNAs play a fundamental role in cancer progression. BBOX1 anti-sense RNA 1 (BBOX1-AS1) is a recently clarified lncRNA and has been identified as dysregulated in various carcinomas, and it contributes to poor survival in cancer patients.

**Methods:** We thoroughly searched six databases for eligible articles published as of 27, April 2023. The association of BBOX1-AS1 expression levels with prognostic and clinicopathological parameters was assessed by odds ratios (OR) and hazard ratios with 95% CIs. Additionally, we further validated our results utilizing the GEPIA online database.

**Results:** Eight studies comprising 602 patients were included in this analysis. High BBOX1-AS1 expression indicated poor overall survival (OS) (hazard ratios = 2.30, 95% Cl [1.99, 2.67], *p* < 0.00001) when compared with low BBOX1-AS1 expression. Furthermore, BBOX1-AS1 expression was positively correlated with lymph node metastasis (OR = 3.00, 95% CI [1.71–5.28], *p* = 0.0001) and advanced tumor stage (OR = 3.74, 95% CI [2.63–5.32], *p* < 0.00001) for cancer patients. Moreover, BBOX1-AS1 was remarkably upregulated in 12 malignancies, and the elevated BBOX1-AS1 expression predicted poorer OS and worse disease-free survival (DFS) confirmed through the GEPIA online gene analysis tool.

**Conclusion:** The findings highlight that BBOX1-AS1 was significantly associated with detrimental overall survival, disease-free survival, lymph node metastasis and tumor stage; thus, it could act as a novel promising biomarker to predict the clinicopathological characteristics and prognosis for various cancers.

## Introduction

With rising morbidity and mortality, cancer has become a serious global health concern, and an additional economic burden is attributable to improving cancer survival worldwide (Ward et al., 2021). The GLOBOCAN 2020 estimated there were nearly 19.3 million new malignant tumors patients and 10.0 million deaths in 2020. Besides, approximately 28.4 million cases will be diagnosed in 2040 (Sung et al., 2021). Currently, cancer patients with lymph node metastasis or advanced tumor stage exhibit a poor prognosis. However, no ideal cancer biomarker can diagnose cancer effectively (Jayanthi et al., 2017). Therefore, a novel biological target capable of early detection and prediction prognosis is urgently required.

Long non-coding RNAs (lncRNAs) are defined as RNAs with longer than 200 nucleotides that lack protein coding potential (Engreitz et al., 2016). Additionally, with the application of nascent transcriptomics technology, abundant molecular mechanisms of lncRNAs have been identified (Nojima and Proudfoot, 2022). Mounting evidence demonstrates that lncRNAs play an indispensable role in cancer progression, tumorigenesis, and immune responses (Bhan et al., 2017; Ebrahimi et al., 2022; Park et al., 2022). For instance, increased lncRNA H19 expression is notably associated with elevated metastasis rates, decreased therapeutic sensitivity and enhanced cancer progression via modulation of numerous targets and molecular pathways, such as RUNX1, STAT3, β-catenin, and FOXM1 in various cancers (Hashemi et al., 2022). Furthermore, high lncRNA KCNQ1OT1 expression correlates with worse overall survival (OS) and affects the CD8^+^ T-cell responses in colorectal tumors (Lin et al., 2021). Therefore, lncRNAs could be developed as novel targets for various cancer diagnostics and drug discovery.

BBOX1 anti-sense RNA 1 (BBOX1-AS1) is a recently clarified lncRNA and has been identified to be dysregulated in several carcinomas, which include lung cancer (Shi et al., 2021; Zhang et al., 2022), cervical cancer (Xu et al., 2020), oral squamous cell carcinoma (OSCC) (Zhao et al., 2022), ovarian cancer (Yao et al., 2021), colorectal cancer (Liu et al., 2022; Shi et al., 2022), nasopharyngeal carcinoma (Jiang et al., 2021), hepatocellular carcinoma (Tao et al., 2021), pituitary adenoma (Wu et al., 2022), and esophageal squamous cell carcinoma (ESCC) (Sheng et al., 2022). Clinically, high BBOX1-AS1 expression is closely associated with poor OS and clinicopathological characteristics, such as tumor stage, lymph node metastasis, tumor size, and differentiation in various malignancies (Lian et al., 2021; Tao et al., 2021; Sheng et al., 2022; Shi et al., 2022). Moreover, BBOX1-AS1 contributes to tumor cell metastasis and proliferation, along with reduces cell apoptosis and ferroptosis via various mechanisms (Pan et al., 2022; Zhang et al., 2022). However, owing to the limited clinical patient sample size, the effect of this relevance is poorly characterized. Thus, we conducted this meta-analysis to evaluate the application of BBOX1-AS1 in the prognosis of various malignancies.

## Materials and methods

### Search strategy

We thoroughly searched six databases, which included SinoMed, Web of Science, EBSCO, Cochrane Library, PubMed, and Ovid for eligible articles published up as of 27 April, 2023. The following search keywords were adopted to identify relevant publications: “long non-coding RNA BBOX1-AS1” OR “BBOX1 anti-sense RNA 1” OR “BBOX1-AS1” OR “lncRNA BBOX1-AS1.” Furthermore, the references in these retrieved articles were also manually searched to examine potentially eligible studies. Only manuscripts written in English or Chinese were included.

### Inclusion and exclusion criteria

Inclusion criteria: 1) BBOX1-AS1 expression was identified in cancerous tissues and corresponding non-cancerous tissues; 2) all patients did not receive preoperative anticancer treatment; 3) the approach of detecting BBOX1-AS1 expression was qRT-PCR; 4) studies reported clinicopathological characteristics or prognostic outcomes such as OS, disease-free survival (DFS), and Kaplan–Meier (K-M) curves to calculate the hazard ratios (HR) (95% CI) indirectly; and 5) patients were divided into high- and low-expression groups, according to BBOX1-AS1 expression levels.

Exclusion criteria: 1) studies absent of clinicopathological characteristics or prognostic outcomes; 2) duplicated articles; 3) case reports, review articles, meta-analysis, editorials, non-human studies and conference reports; and 4) studies based on the public database.

### Data extraction and quality assessment

After reviewing all the eligible articles, two investigators (GYL and YZW) independently extracted the requisite data, and all disagreements were resolved by discussing with two additional reviewers (LD and TY). The necessary information was carefully extracted from each study: author’s last name, publication year, cancer types, total number of patients, the number of patients in the high- and low-BBOX1-AS1 expression groups, detection methods, outcomes, the HR and corresponding 95% CIs for OS, follow-up time, and extraction methods for OS data. The HR (95% CI) for OS was calculated using the Engauge Digitizer 4.1 software indirectly through K-M curves if the eligible articles failed to offer the HR (95% CI) directly (Tierney et al., 2007). If both univariate and multivariate methods were performed in analyzing the data, the latter was preferred.

### Validation of bioinformatics database

We compared the differences between BBOX1-AS1 expression in various human cancerous and non-cancerous tissues. Furthermore, we validated its prognostic value utilizing the GEPIA online database based on the TCGA and GTEx data (http://gepia.cancer-pku.cn/) (Tang et al., 2017; Li et al., 2021). Survival plots of the association with BBOX1-AS1 expression and DFS or OS were displayed as K-M curves derived from different cancer data sets. The median was established for the cut-off value. All *p*-value <0.05 was defined as statistically significant.

### Statistical analysis

Data management was conducted with the EndNote 20.2 software. All analysis were carried out with the Review Manager 5.3 software. The effect of BBOX1-AS1 levels on OS, tumor differentiation, lymph node metastasis, tumor stage, tumor size, patient age, and gender were assessed by odds ratio (OR) and HRs with 95% CIs. Cochrane Higgins I^2^ statistics was applied to calculate heterogeneity among all included studies. The fixed-effects model was used for data analysis if heterogeneity was absent (I^2^< 50%). Otherwise, the more appropriate random-effects model was adopted. A *p*-value <0.05 was considered to be statistically significant.

## Results

### Included articles

**FIGURE 1:**
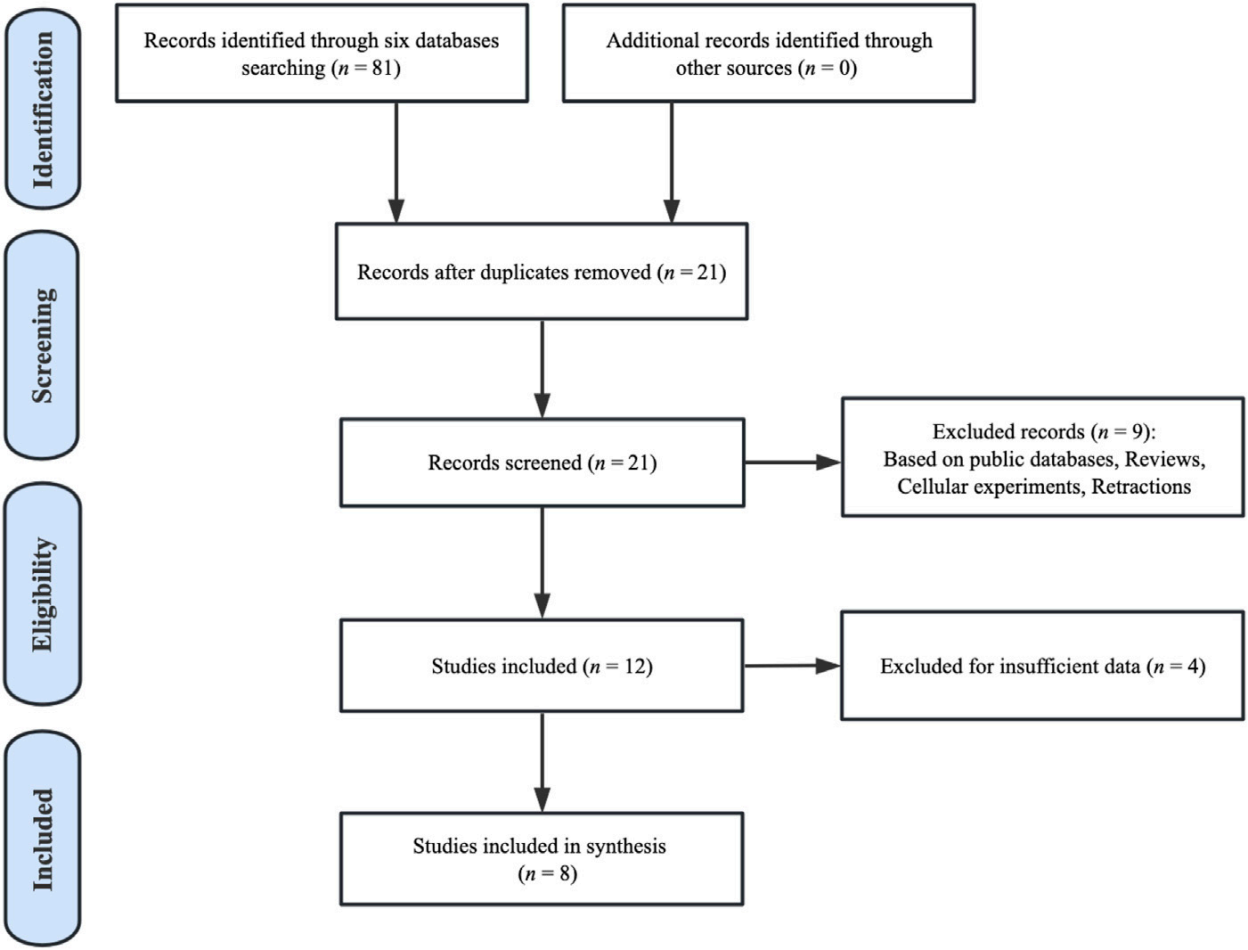
Article selection flowchart.

By searching six databases (up to 27 April 2023), a total of 81 publications concerning the prognosis of BBOX1-AS1 and cancer patients were retrieved preliminarily. Among the 81 pieces of literature, 60 duplicate studies were excluded and 21 studies were selected for abstract screening. Nine publications were excluded since they were retraction articles, cell-based experiments, reviews, or research studies based on public databases. Then, we carefully removed another four studies because they lacked sufficient data regarding clinicopathologic characteristics or prognosis for analysis. Finally, eight studies from 2020 to 2023 were considered eligible for the meta-analysis. The selection flowchart for the included publications is depicted in Figure 1.

### Study characteristics

These studies were published between 2020 and 2023, and the sample sizes ranged from 40 to 135. A total of 602 patients were divided into the low- and high-BBOX1-AS1 expression groups, with 299 and 303 cases in each group, respectively. The studies were performed in different regions of China and six types of malignancies were examined: lung cancer, cervical cancer, ESCC, gastric cancer, esophageal carcinoma, and hepatocellular carcinoma. Among these included studies, seven estimated the correlation between BBOX1-AS1 and OS of patients. Table 1 summarizes the detailed characteristics of these included studies.

**TABLE 1:** Main characteristics of included studies.

Author	Cancer type	Sample size (n)	High expression(n)	Low expression(n)	Detection method	Outcome	HR (95% CI) for OS	Follow-up (m)	Data extraction method
Xu et al. (2020)	CC	100	50	50	qRT-PCR	CP, OS	2.15 (1.08–4.29)	60	Indirectly
Shi et al. (2021)	NSCLC	76	38	38	qRT-PCR	CP, OS	2.57 (1.06–6.19)	60	Indirectly
Lian et al. (2021)	NSCLC	135	68	67	qRT-PCR	CP, OS	2.07 (1.05–4.06)	60	Directly
Pan et al. (2022)	ESCC	78	39	39	qRT-PCR	CP, OS	2.13 (1.05–4.33)	60	Indirectly
Sheng et al. (2022)	ESCC	45	23	22	qRT-PCR	CP, OS	3.02 (1.33–6.86)	70	Directly
Cai et al. (2022)	GC	40	21	19	qRT-PCR	CP	NA	NA	NA
Ma et al. (2023)	EC	45	22	23	qRT-PCR	CP, OS	3.02 (1.33–6.86)	60	Directly
Tao et al. (2022)	HC	83	42	41	qRT-PCR	CP, OS	1.49 (0.53–4.21)	60	Indirectly

Abbreviations: CC, cervical cancer; NSCLC, non–small-cell lung cancer; ESCC, esophageal squamous cell cancer; GC, gastric cancer; EC, esophageal carcinoma; HC, hepatocellular carcinoma; qRT-PCR, quantitative real-time reverse-transcription polymerase chain reaction; CP, clinicopathologic parameters; OS, overall survival; HR, hazard ratio; CI, confidence interval; NA, not available.

### Association between BBOX1-AS1 expression and clinical covariates

**FIGURE 2:**
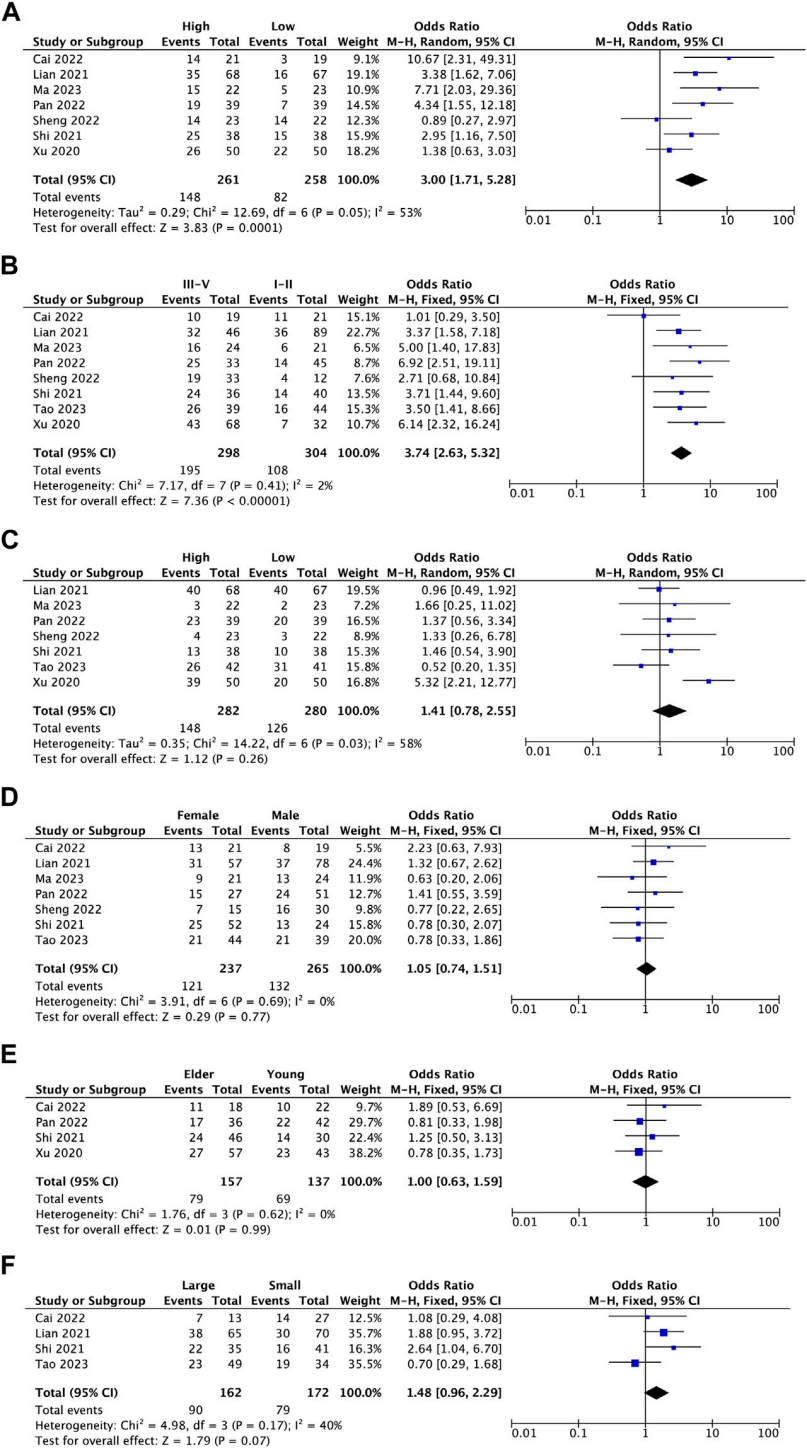
Forest plots assessing the association between BBOX1-AS1 expression and clinicopathological parameters [**(A)** lymph node metastasis, **(B)** stage, **(C)** differentiation, **(D)** gender, **(E)** age, and **(F)** tumor size].

This meta-analysis examined the association between BBOX1-AS1 expression and clinical covariates. The results revealed that BBOX1-AS1 overexpression was remarkably correlated with lymph node metastasis (I^2^ = 53%, *p* = 0.0001) (Figure 2A) and more advanced tumor stage (I^2^ = 2%, *p* < 0.00001) (Figure 2B). However, BBOX1-AS1 expression did not associate with tumor differentiation (I^2^ = 58%, *p* = 0.26) (Figure 2C) and patient gender (I^2^ = 0%, *p* = 0.77) (Figure 2D). Subsequently, as for patient age and tumor size, the meta-analysis was performed stratified by a cut-off values such as age ≥ 60 years (elder) *versus* <60 years (young) and tumor size ≥ 5 cm (large size) *versus* <5 cm (small size). The pooled results demonstrated high BBOX1-AS1 expression was not significantly associated with patient age (I^2^ = 0%, *p* = 0.99) (Figure 2E) and tumor size (I^2^ = 40%, *p* = 0.07) (Figure 2F).

### Association between BBOX1-AS1 expression and overall survival

**FIGURE 3:**
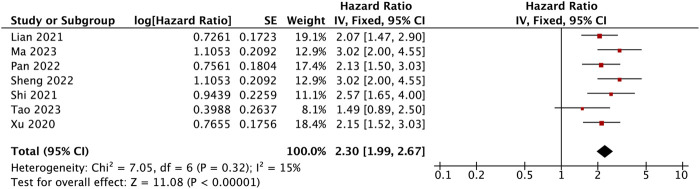
Forest plot for the association between BBOX1-AS1 expression with OS.

Seven studies with data from 562 patients reported the association between BBOX1-AS1 as a prognostic biomarker of malignancies and OS. Pooled HR was 2.30 (95% Cl: 1.99, 2.67, *p* < 0.00001). A fixed-effects model was applied to evaluate the HR of these studies because of low heterogeneity (I^2^ = 15%). The result suggested that the upregulation of BBOX1-AS1 predicted poor OS among various malignancies (Figure 3). Furthermore, the subgroup analysis was carried out, based on cancer types, sample sizes, and extracted methods. As presented in Table 2, when compared with low BBOX1-AS1 expression in these subgroup analysis, high BBOX1-AS1 expression revealed poor OS in cancer patients, regardless of the cancer types, sample sizes, and extracted methods (*p* < 0.00001). A fixed-effects model was utilized since all these stratified analysis manifested low or no heterogeneity.

**TABLE 2:** Subgroup analysis of the relationship between BBOX1-AS1 expression and OS.

Variable	Total cases (n)	HR 95% CI	*p*	I^2^ (%)	Model
Overall survival	562	2.30 [1.99, 2.67]	<0.00001	15	Fixed
Cancer types					
NSCLC	211	2.24 [1.71, 2.93]	<0.00001	0	Fixed
ESCC	168	2.62 [2.10, 3.28]	<0.00001	11	Fixed
Other cancers	183	1.92 [1.44, 2.56]	<0.00001	25	Fixed
Sample size					
≥80	318	1.98 [1.59, 2.46]	<0.00001	0	Fixed
<80	244	2.61 [2.14, 3.19]	<0.00001	0	Fixed
Extracted method					
Indirectly	337	2.10 [1.73, 2.57]	<0.00001	0	Fixed
Directly	225	2.57 [2.06, 3.20]	<0.00001	28	Fixed

Abbreviations: NSCLC, non–small-cell lung cancer; ESCC, esophageal squamous cell cancer.

### Validation of results based on TCGA

**FIGURE 4:**
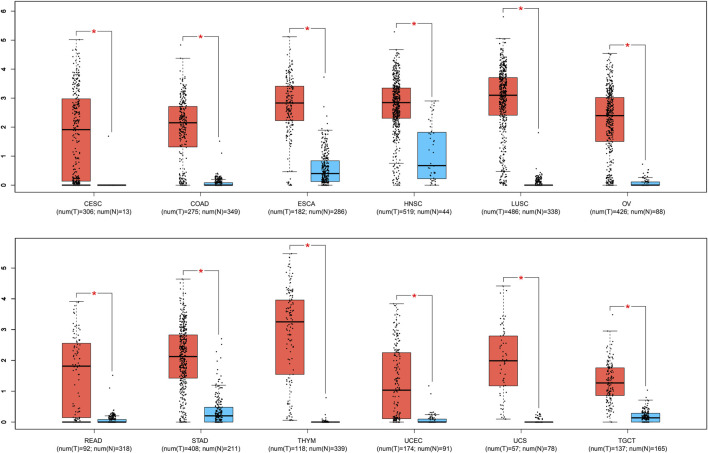
BBOX1-AS1 expression in 12 types of tumor tissues (red) vs. normal tissues (blue). “*” *p* < 0.01. Abbreviations: T, tumor tissues; N, normal tissues; CESC, cervical squamous cell carcinoma and endocervical adenocarcinoma; COAD, colon adenocarcinoma; ESCA, esophageal carcinoma; HNSC, head and neck squamous cell carcinoma; LUSC, lung squamous cell carcinoma; OV, ovarian serous cystadenocarcinoma; READ, rectum adenocarcinoma; STAD, stomach adenocarcinoma; THYM, thymoma, UCEC, uterine corpus endometrial carcinoma; UCS, uterine carcinosarcoma; TGCT, testicular germ cell tumors.

**FIGURE 5:**
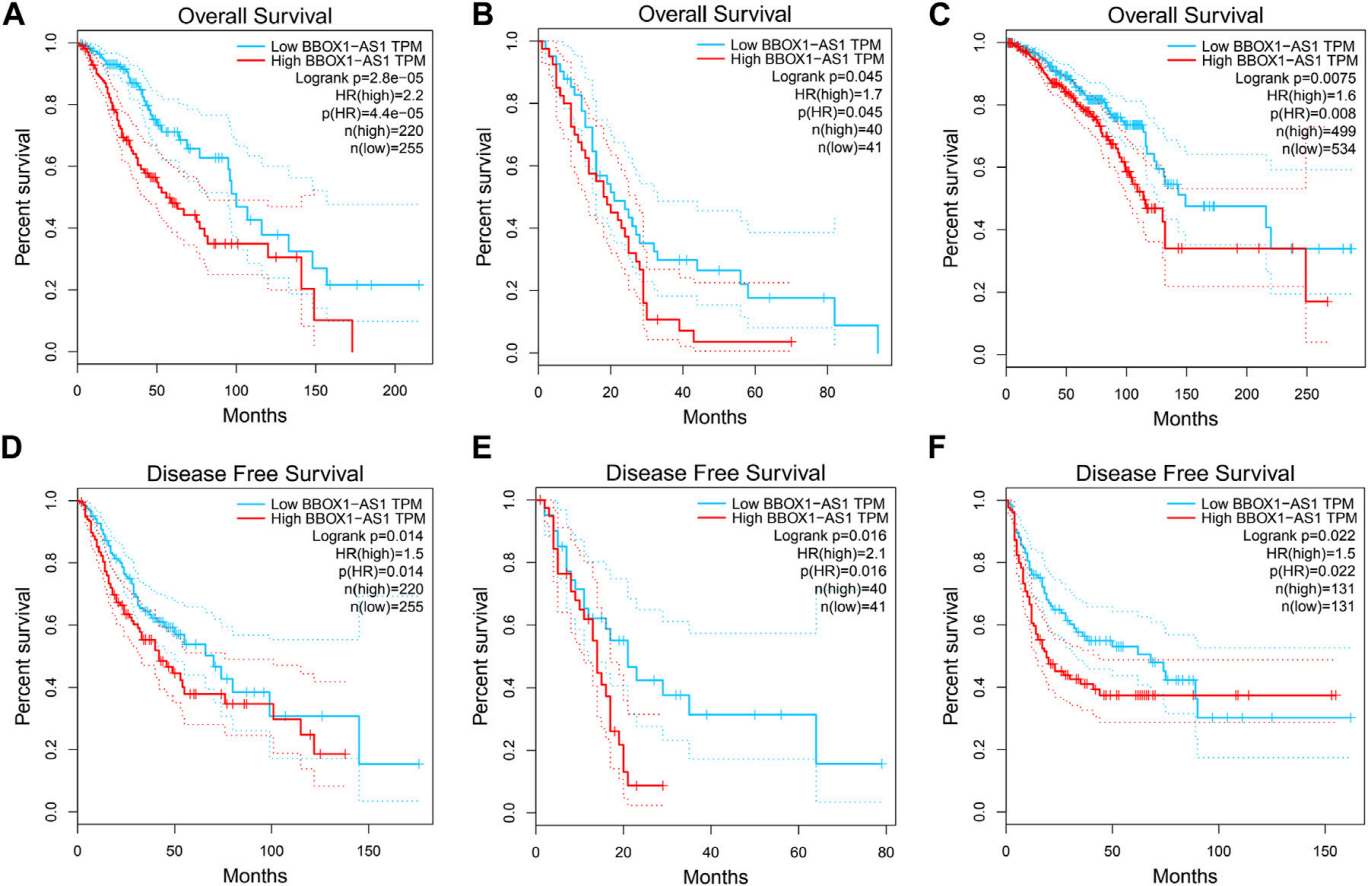
Verification of the prognostic value of BBOX1-AS1 in the TCGA database. **(A)** OS plots of BBOX1-AS1 in LGG, **(B)** OS plots of BBOX1-AS1 in MESO, **(C)** OS plots of BBOX1-AS1 in BRCA, **(D)** DFS plots of BBOX1-AS1 in LGG, **(E)** DFS plots of BBOX1-AS1 in MESO, and **(F)** DFS plots of BBOX1-AS1 in SARC. Abbreviations: TCGA, The Cancer Genome Atlas; LGG, brain lower grade glioma; MESO, mesothelioma; BRCA, breast invasive carcinoma; SARC, sarcoma.

To further validate the significance of BBOX1-AS1 in diverse cancers, the GEPIA online gene analysis tool was utilized. Figure 4 shows that the overexpression of BBOX1-AS1 was identified in 12 malignancies. In addition, the connection between BBOX1-AS1 expression and prognosis with various malignancies was verified with K-M curves. Similar to our meta-analysis, the upregulated BBOX1-AS1 expression was dramatically correlated to a poorer OS in three malignancies, which included brain lower grade glioma (LGG), mesothelioma (MESO), and breast invasive carcinoma (BRCA) (*p* < 0.05) (Figures 5A–C). Besides, the overexpression of BBOX1-AS1 was negatively related to a poorer DFS in LGG, MESO, and sarcoma (*p* < 0.05) (Figures 5D–F).

## Discussion

Recently, a burgeoning number of studies discovered that lncRNAs were dysregulated in diverse human malignancies. Many may act as novel potential markers for early diagnosis, therapy, and prediction of prognosis in cancers. Hence, in recent years, numerous reviews, clinical trials, basic experiments, and systematic review publications, such as our meta-analysis, have been performed to gradually demonstrate the correlation between lncRNAs and diverse tumor prognoses (Shen et al., 2018; Tan et al., 2021; Li et al., 2022). Furthermore, numerous previous studies investigated that BBOX1-AS1 may serve as a novel lncRNA to advance the development of cancers through various biological mechanisms including cell migration, invasion, proliferation, and apoptosis inhibition (Liao et al., 2020; Xu et al., 2020; Shi et al., 2021). Moreover, elevated BBOX1-AS1 may negatively interact with the miRNAs expression, such as miR-27a-5p (Shi et al., 2021), miR-361-3p (Xu et al., 2020; Lian et al., 2021; Yao et al., 2021; Wu et al., 2022), miR-3940-3p (Jiang et al., 2021; Liu et al., 2022; Zhao et al., 2022), miR-513a-3p (Pan et al., 2022), and miR-125b(a)-5p (Wang et al., 2021) in multiple cancers. For example, Sheng et al. (2022) proved that the upregulation of BBOX1-AS1 could accelerate ESCC progression by activating the Wnt/β-catenin pathway via promoting HOXB7 expression. In the respiratory system, Shi et al. (2021) reported that BBOX1-AS1 played tumor-promoting roles in non–small-cell lung cancer by activating the MELK/FAK signaling axis. In addition, BBOX1-AS1 could boost cell migration, proliferation, and the malignant phenotype by upregulating LAMC2 expression in OSCC (Zhao et al., 2022). Collectively, these studies demonstrated that BBOX1-AS1 acted a vital role in human malignancies' development and progression. Nevertheless, the prognostic effect of BBOX1-AS1 in clinical application was limited by the small sample capacity. To the best of our knowledge, no meta-analysis has been performed to investigate the effects of BBOX1-AS1 expression on the prognosis of patients with malignancy.

In this meta-analysis, we identified the relevance between BBOX1-AS1 expression and human malignancies’ prognostic parameters. The results illustrated that an increase in BBOX1-AS1 was dramatically related to unfavorable OS (HR = 2.30, 95% CI: 1.99–2.67, *p* < 0.00001). Furthermore, subgroup analysis demonstrated the same result despite cancer types, sample sizes, and extracted methods. Additionally, high BBOX1-AS1 expression significantly was connected with lymph node metastasis (OR = 3.00, 95% CI: 1.71–5.28, *p* = 0.0001) and more advanced tumor stage (OR = 3.74, 95% CI: 2.63–5.32, *p* < 0.00001) in cancer patients. Clinically, a study involving 10,126 cases demonstrated that lymph node metastasis was tightly correlated with a high incidence of regional recurrence and distant metastasis, which directly lowered the survival rate of cancer patients (Zhang et al., 2019). Besides, mounting evidence have suggested that advanced tumor stage tended to have worse prognosis, which includes shorter progression-free survival (PFS), OS, and post-progression survival (PPS) (Fallowfield and Fleissig, 2011; Ogasawara et al., 2016). However, no remarkable association was detected between high BBOX1-AS1 expression and tumor differentiation (OR = 1.41, 95% CI: 0.78–2.55, *p* = 0.26), patient age (OR = 1.00, 95% CI: 0.63–1.59, *p* = 0.99), patient gender (OR = 1.05, 95% CI: 0.74–1.51, *p* = 0.77), and tumor size (OR = 1.48, 95% CI: 0.96–2.29, *p* = 0.07). Meanwhile, the GEPIA database was applied to confirm our results as extensively as possible. Elevated BBOX1-AS1 expression levels were also established in 12 different types of malignancies. Moreover, increased BBOX1-AS1 expression was associated with poor OS in LGG, MESO, and BRCA and with worse DFS in LGG, MESO, and sarcoma. Taken together, this consequence demonstrated that BBOX1-AS1 could be applied as a novel biomarker for prognosis or detection of various malignancies.

Nevertheless, several limitations in this study should be noted as well. First, as a novel biomarker, the earliest study demonstrating the association between BBOX1-AS1 and cancers was published in 2020. Although we thoroughly searched six databases, only eight studies were finally enrolled for this analysis after careful screening. This may hence be one of the explanations for the relatively small amount of literature included in our study. However, the GEPIA database and subgroup analysis were adopted to strengthen our results, and they demonstrated that our results were reliable. Second, therapeutic regimens may also be considered to play a vital role in cancer patient survival. Thus, different treatments, to some extent, may impact the calculation of HR or OR values. Third, there was moderate heterogeneity in some analyses such as lymph node metastasis (I^2^ = 53%) and tumor differentiation (I^2^ = 58%). Since all included studies were from different hospitals and examination approaches used by individual studies might not be uniform, these may likely contributed to heterogeneity. Consequently, an appropriate random-effects model had to be utilized, which might somewhat degrade the accuracy of results. Fourth, studies published only in English or Chinese were included, whereas studies exploring the relationship between BBOX 1-AS 1 and survival in cancer patients in other languages were omitted. Fifth, some HRs were extracted indirectly through rebuilding the K-M curves, which might inevitably cause possible deviations. Notwithstanding the intrinsic deficiencies, our results render robust evidence that overexpressed BBOX1-AS1 indicates poor prognosis in cancer patients.

## Conclusion

In conclusion, our meta-analysis demonstrated that overexpressed BBOX1-AS1 is exceedingly correlated with worse OS, DFS, lymph node metastasis, and advanced tumor stage in carcinomas. Specifically, BBOX1-AS1 can be served as a novel promising biomarker for predicting the clinicopathological characteristics and prognosis for various cancers. Furthermore, *in vitro*/*in vivo* validation of the promotion of BBOX1-AS1 in different malignancies and its effects on tumor pathology could be investigated in future studies.

## Data Availability

The original contributions presented in the study are included in the article further inquiries can be directed to the corresponding author.
